# Green and Heavy-Duty Anticorrosion Coatings: Waterborne Epoxy Thermoset Composites Modified through Variation of Zinc Dust Loading and Incorporation of Amine-Capped Aniline Trimer and Graphene Oxide

**DOI:** 10.3390/polym16091252

**Published:** 2024-04-30

**Authors:** Yun-Xiang Lan, Yun-Hsuan Chen, Ying-Lung Chao, Yu-Hsuan Chang, Yu-Chi Huang, Wei-Ren Liu, Wei-Tsan Wong, Andrew Chi-Fa Sun, Karen S. Santiago, Jui-Ming Yeh

**Affiliations:** 1 Department of Chemistry, Center for Nanotechnology at Chung Yuan Christian University, Chung Li 32023, Taiwan, China; lanyunxiang@cycu.org.tw (Y.-X.L.); g11163016@cycu.edu.tw (Y.-H.C.); g11163023@cycu.edu.tw (Y.-L.C.); s10913225@cycu.edu.tw (Y.-H.C.); s10913206@cycu.edu.tw (Y.-C.H.); 2 Department of Chemical Engineering, R&D Center for Membrane Technology, Center for Circular Economy, Chung Yuan Christian University, Taoyuan City 32023, Taiwan, China; 3 Shiny Chemical Industrial Co., Ltd., Kaohsiung 82841, Taiwan, China; wayne_wong@shinychem.com.tw (W.-T.W.); andrewsun@shinychem.com.tw (A.C.-F.S.); 4 Department of Chemistry, College of Science, Research Center for the Natural and Applied Sciences, University of Santo Tomas, Manila 1015, Philippines; kssantiago@ust.edu.ph

**Keywords:** waterborne, epoxy, electroactive, GO, corrosion, heavy-duty

## Abstract

In this study, an array of environmentally friendly and heavy-duty anticorrosion composite coatings were prepared. The synthesis involved amine-capped aniline trimer (ACAT) produced by an oxidative coupling reaction and graphene oxide (GO) prepared based on Hummer’s method, and later, the waterborne epoxy thermoset composite (WETC) coatings were prepared by thermal ring-opening polymerization of EP 147w, a commercial waterborne epoxy resin, in the presence of ACAT and/or GO with zinc dust (ZD). A synergistic effect was observed by replacing a significant amount of the ZD loading in the WETC by simultaneously incorporating a small amount of ACAT and GO. The electrochemical corrosion measurements of the as-prepared WETC coatings indicated that incorporating 5% *w*/*w* ACAT or 0.5% *w*/*w* GO separately replaced approximately 30% *w*/*w* or 15% *w*/*w* of the ZD, respectively. Moreover, the WETC coatings containing 5% *w*/*w* ACAT and 0.5% *w*/*w* GO simultaneously were found to replace 45% *w*/*w* of the ZD. A salt spray test based on ASTM B-117 also showed a consistent trend with the electrochemical results. Incorporating small amounts of ACAT and GO in WETC coatings instead of ZD not only maintains the anticorrosion performance but also enhances adhesion and abrasion resistance, as demonstrated by the adhesion and abrasion tests.

## 1. Introduction

Globally, metal corrosion leads to annual economic losses approximated at around USD 22 trillion, amounting to ~3% of the world’s Gross Domestic Product [1,2]. Several researchers have actively devoted extensive efforts to studying metal corrosion. Conventionally, the epoxy resin (ER) coating utilized for corrosion resistance is frequently a solvent-based matrix, which contains a defined concentration of volatile compounds (VOCs) that exert profound deleterious effects on human beings and environmental ecosystems. Prompted by the imperative to safeguard human health and carry out conscientious environmental stewardship, the utilization of ERs necessitates a paradigm shift, transitioning from conventional solvent-based modalities to ecologically benign waterborne systems [3,4]. The undeniable reality persists that, notwithstanding the commercialization of waterborne ER coatings for more than four decades, their long-term anticorrosion efficacy remains sub-optimal owing to inherent deficiencies in impeding the ingress of water and corrosive ions, which is attributable to their inadequate barrier properties [4]. Attempts to enhance the anticorrosive features of organic coatings through diverse methods have been recorded. Distinctive nanofillers were investigated, including SiO_2_ [5,6], polyaniline [7,8], graphene [9,10], and hexagonal boron nitride (h-BN) [4,11].

Among the various methods employed against corrosion, zinc-rich ER coatings are extensively utilized for protecting steel materials due to their remarkable qualities such as superior adhesion, corrosion resistance, and mechanical performance [12,13,14,15,16]. It is worth mentioning that these coatings possess distinct corrosion protection mechanisms, including cathodic protection and barrier effects [17,18,19]. In the initial stages of corrosion, cathodic protection plays a crucial role in defense. When corrosive agents penetrate the coating, it creates an electrochemical cell system, in which zinc particles act as sacrificial anodes, while the steel substrate serves as the cathode, safeguarding the steel substrate. However, in the mid- to late stages of corrosion, the corrosion by-products of zinc accumulate within the micro-pores of the coating [20]. This delay in the infiltration of the corrosive medium into the coating enhances the coating’s shielding effect [21,22,23].

To ensure effective cathodic protection of coatings, the formulation of a coating composition is often altered by increasing the zinc content to exceptionally high levels (~80% *w*/*w*), [24,25]. However, this approach comes with a physical property trade-off, leading to a decline in the mechanical performance of the coating and simultaneously increasing production costs. Consequently, researchers have shifted their focus towards using various forms of zinc particles [26] or incorporating different conductive materials to mitigate the need for high zinc concentrations in the coating. For example, Hu Wang et al. [27] observed that adding 0.50% gold nanoparticles yielded the most significant reinforcement effect on coatings with a 50% zinc content, surpassing those with a zinc content as high as 80% *w*/*w*. Similarly, Qingjun Zhu et al. [28] found that substituting lamellar metal powder for spherical zinc pigments enhanced the electrical connectivity between metal particles, extended the path for corrosive substances to penetrate the coating, and bolstered the coating’s physical shielding capabilities, all while reducing the pigment content by one-third.

Nonetheless, these conductive materials are characterized by their relatively high cost, rendering them unsuitable for large-scale industrial production. Furthermore, numerous attempts have been made to develop polymer composites modified by nanofillers to enhance the tribological properties of the matrix. This is because polymer composites offer excellent manufacturability and exceptional wear resistance, holding immense potential for replacing metallic materials in tribological applications. As epoxy coatings adhere to metal surfaces through chemical bonding, active epoxy functional groups such as hydroxyls tend to form hydrogen bonds with the surface-active substrate. Studies have shown that adding GO at a concentration of 0.5 wt% significantly enhances wear resistance in epoxy resin, reducing the wear rate by 90.0–94.1% compared to pure epoxy resin [29]. Other research indicates that the addition of functionalized GO increases the number of epoxy functional groups and enhances hydrogen bonding, thereby improving adhesion [30]. Further functionalization of GO particles helps reduce the free surface energy at the polymer–nanofiller interface, enhancing adhesion and thereby improving the physical and mechanical properties of the composite material [31]. Additionally, traditional solvent-based epoxy resins, when used in engineering applications, give rise to substantial emissions of volatile organic compounds (VOCs), resulting in significant environmental pollution and potential health hazards [32,33]. Currently, waterborne coatings, which primarily employ water as their solvent, have emerged globally as the most prominent category of coatings for environmental protection purposes [34]. By eliminating or reducing the use of volatile organic compounds (VOCs) [35], waterborne coatings are reported to offer distinctive advantages such as low toxicity [36], non-flammability [37], and outstanding adhesion to metals, which then present a wide range of application possibilities [38,39].

In previous studies, reduced graphene oxide (rGO) and aniline oligomer (ACAT) have been utilized to reduce the zinc content in zinc-rich coatings, albeit with solvent-based resins [40,41].

Recognizing environmental concerns, this study will employ waterborne epoxy thermoset (WET) materials as the key coating substrate. Subsequently, small amounts of ACAT and GO will be prepared, characterized, and attempted to be incorporated into waterborne epoxy thermoset composite (WETC) coatings to replace a considerable amount of zinc dust (ZD) for the application of heavy-duty anticorrosion coating. It is anticipated that the incorporation of small amounts of ACAT and GO in a WETC to replace a significant amount of ZD may not only maintain a similar anticorrosion performance of the WETC coating but also enhance its physical properties, such as adhesion or wear resistance. The anticorrosion performance of the prepared WETC coatings will be evaluated through a series of electrochemical corrosion measurements and industrial salt spray testing (ASTM B-117 [42]). Moreover, adhesion testing and wear resistance testing of WETC coatings will be conducted based on the evaluation of ASTM D3359 [43] and ASTM D4060 [44], respectively.

This research represents a novel approach by employing waterborne epoxy thermoset materials and integrating ACAT and GO to develop heavy-duty anticorrosion coatings with reduced zinc contents. By replacing ZD with ACAT and GO, this study aims to not only address environmental concerns but also enhance the physical and anticorrosive properties of the coatings. The utilization of waterborne epoxy thermoset materials adds to the novelty of the research, offering a potential for eco-friendly and high-performance coating solutions in various industrial applications.

## 2. Materials and Methods

### 2.1. Chemicals and Instrumentation

Zinc dust (ZD) was provided by Shiny Chemical Industrial Co., Ltd. (Kaohsiung, Taiwan), Graphite powder was purchased from Sigma-Aldrich (St. Louis, MI, USA). Non-crystallizing Bisphenol A/F liquid epoxy resin (BECKOPOX™ EP 147w; ALLNEX, Frankfurt, Germany) and waterborne acetic-acid-free aliphatic polyamine adduct (BECKOPOX™ EH 613w/80WA; ALLNEX, Frankfurt, Germany) were used as received without further treatment. Ammonium hydroxide solution (NH_4_OH, Riedel-de Haën, 25%, Charlotte, NC, USA), N, N-dimethyl-acetamide (DMAc, Duksan, 99%, Gyeonggi-do, Republic of Korea), ammonium persulfate ((NH_4_)_2_S_2_O_8_, APS, J. T. Baker, 98%, Phillipsburg, NJ, USA), Toluene (J. T. Baker, 99.5%, NJ, USA), potassium carbonate (K_2_CO_3_, J. T. Baker, >99%, NJ, USA), sulfuric acid (H_2_SO_4_; Fluka, 99.0%, Selzer, Germany), sodium chloride (NaCl; Aldrich, 99.0%, St. Louis, MI, USA), sodium nitrate (NaNO_3_; Fluka, 99.5%, Selzer, Germany), potassium permanganate (KMnO_4_; J.T.Baker, 99.0%, NJ, USA), hydrochloric acid (HCl; Fluka, 37%, Selzer, Germany), and hydrogen peroxide (H_2_O_2_; First Chemical Co., Ltd., 35%, Taipei, Taiwan) were used. All of the chemicals were of analytical reagent grade unless otherwise stated. CRS with a diameter of 18.2 mm and thickness of 1.177 mm was used as the electrochemical electrode. Carbon steel (CS) with dimensions of 10 cm × 7 cm × 0.8 mm was used for the salt spray test. Both CRS and CS were provided by Shiny Chemical Industrial Co., Ltd. (Kaohsiung, Taiwan).

The XRD patterns of ZD and GO/rGO were recorded with Cu Ka (λ_l_ = 0.15418 nm) by using a Philips X’pert Pro (Amsterdam, The Netherlands). The morphological structure was observed under a field-emission scanning electron microscopy (SEM) system (JEOL JSM-7600F, Tokyo, Japan). The FTIR spectra of materials and samples were collected using an FTIR spectrometer (Bio-rad FTS-7, Hercules, CA, USA) at room temperature. Attenuated total reflectance FTIR spectra were collected at a resolution of 4.0 cm^–1^. The chemical structure of the material was identified using hydrogen signals through a liquid nuclear magnetic resonance instrument (Bruker NMR 400, Billerica, MA, USA). A 5 mg sample was dissolved in methanol and filtered with a 0.45 um pore size filter, and then, UHPLC-qTOFMS (Bruker/Dionex Ultimate 3000, Billerica, MA, USA) was used to identify the molecular weight of the material. We used a UV-Vis Spectrometer (JASCO V-750, Hachioji City, Tokyo) to observe the changes in the UV–visible light absorption of the electroactive aniline trimer. Drop the sample solution intended for cyclic voltammetry (CV) onto a 1 cm^2^ conductive surface of an Indium Tin Oxide Glass (ITO Glass) slide and solidify it. After solidification, immerse it into a 1 M sulfuric acid solution, utilizing a mercury–silver electrode as the standard reference electrode, and a platinum foil as the auxiliary electrode. Scan the potential within the range of −200 to +1000 mV at a scan rate of 1 mV/s to investigate whether the material exhibits redox properties. An iHR320 imaging spectrometer (Horiba Jobin Yvon, Paris, France) at a laser excitation of 532 nm was used to collect Raman scattering spectra. The CA of water droplets was measured using an optical First Ten Angstroms FTA 125 (Portsmouth, VA, USA) goniometry at room temperature. Water droplets of 4 µL were carefully dropped onto the surface of the samples. CA was measured five times at similar locations from the sample surface, and the average was taken as the result. VoltaLab 40 potentiostat/galvanostat was used to test the electrochemical corrosion characteristics of the coating materials within a standard cell equipped with two graphite rods of a counter electrode and a saturated calomel electrode (SCE), along with a working electrode. Electrochemical impedance spectroscopy (EIS) data were recorded on an AutoLab (PGSTAT302 N, Metrohm AG, Herisau, Switzerland) potentiostat/galvanostat electrochemical analyzer. A planetary mixer (Kakuhunter, SK-300SII, Kyoto, Japan) was used to mix the epoxy precursor, hardener, and different types of fillers. A salt spray tester fabricated by Ten Billion Technology Co., Ltd. (Tainan, Taiwan), was used to perform accelerated salt spray corrosion assays of scrapped test panels to evaluate the corrosion resistance according to ASTM B-117. A wear resistance tester (Taber Abrasion Tester) was used to verify the degree of wear resistance of the sample according to ASTM D4060.

### 2.2. Preparation of Amino-Capped Aniline Trimer (ACAT) [45]

**Scheme 1:**
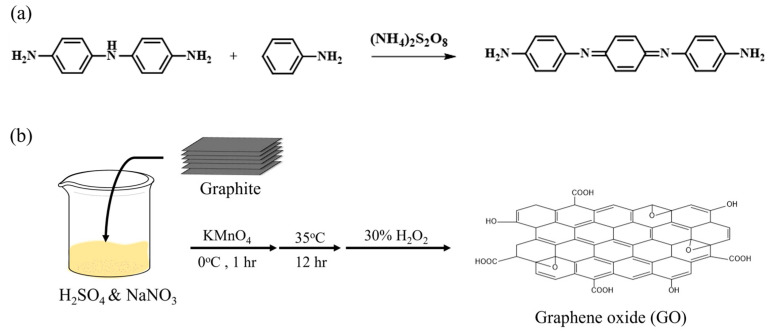
(**a**) Preparation of amine-capped aniline trimer (ACAT) by oxidative coupling reaction. (**b**) Preparation of graphene oxide (GO) by Hummer’s method.

An total of 4.73 g 4,4′-Diaminodiphenylamine sulfate hydrate was dissolved in 150 mL of 1.28 M NaCl solution. The mixture was transferred to an ice bath container. Subsequently, 1.58 g aniline was gradually dissolved in the mixture with vigorous stirring until completely dissolved. APS (7.2 g), pre-dissolved in 50 mL of 1.28 M NaCl, was added to the mixture whilst maintaining vigorous stirring for 1 h. The mixture was filtered and washed several times with 1.0 N HCl and then 1.0 N NH_4_OH. The filtered mixture was poured into 400 mL of 1.0 N NH_4_OH with energetic stirring for 24 h, then re-washed and filtered until neutralized. The mixture was dried at 50 °C in vacuum conditions for 6 h. The preparation process of ACAT is shown in Scheme 1a.

### 2.3. Preparation of Graphene Oxide (GO)

GO was prepared according to a typical procedure [46,47,48]. Briefly, GO derived from SFG44 graphite powder (TIMCAL^®^) was successfully prepared by Hummer’s method through using NaNO_3_/H_2_SO_4_/KMnO_4_/H_2_O_2_, washing, and adjusting the pH to ~5. To begin, 100 mL of sulfuric acid and 1.0 g of sodium nitrate were combined and stirred for 10 min. Following this, 1.0 g of graphite powder was introduced into the mixture while stirring magnetically. Subsequently, 8 g of potassium permanganate was slowly added to the solution, which was stirred for 6 h until it turned dark green. To remove excess KMnO4, hydrogen peroxide was added dropwise while stirring for 10 min. The resulting precipitate was washed multiple times with excess water until the pH of the wash water reached neutral. Finally, the GO precipitate was dried in a vacuum oven at 70 °C for 24 h. The preparation process of GO is shown in Scheme 1b.

### 2.4. Preparation of Waterborne Epoxy Thermoset Coatings (WETCs)

The procedure for preparing the WETC was given as follows: 0.5 g epoxy resin (EP 147w) and 0.375 g curing agent (EH613w) were dissolved in 1 mL of DI water and stirred for 2 h. Subsequently, an appropriate amount of as-prepared mixing solution was cast onto a cold-rolled steel (CRS) coupon with dimensions of 1 cm × 1 cm, followed by curing in a programmed furnace. The programmed heating condition was set to rise from room temperature to 50 °C in 5 min, followed by increasing the temperature up to 120 °C within 1.5 h and maintaining the sample at 120 °C for 4 h.

### 2.5. Preparation of Waterborne Epoxy Thermoset Composite Coatings Containing 1, 3, and 5 wt-% of ACAT with ZD (EWZ), Respectively

**Scheme 2:**
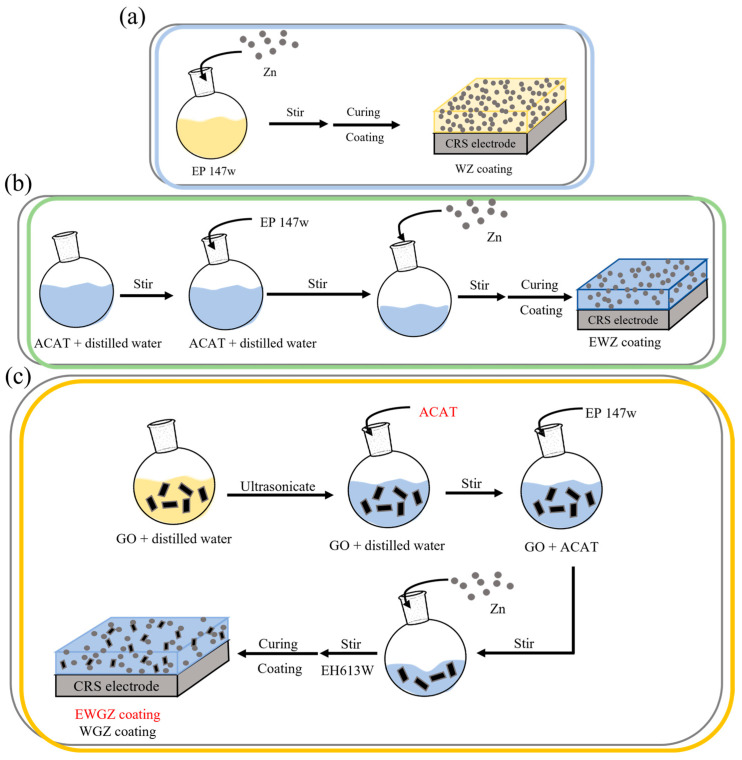
Flowchart for the preparation of distinctive composite coatings of (**a**) WZ, (**b**) EWZ coatings, and (**c**) WGZ and EWGZ.

A specific amount of ACAT was dissolved in 1 mL of DI water, and then continuously stirred for 12 h (refer to Table 1). Subsequently, 0.5 g of EP 147w was introduced into the sample bottle under magnetic stirring for another 12 h. Then, EH613w was incorporated into the sample bottle under magnetic stirring for 2 h. Afterwards, different weight ratios of ZD were introduced into the sample bottle under magnetic stirring for 6 h (refer to Table 1). The as-prepared mixing solution was then coated uniformly onto the CRS coupon, followed by placing the CRS coupon into a programmed furnace. After the curing process, the WETCs containing 1, 3, and 5% *w*/*w* ACAT with ZD (EWZ) were obtained. The flowchart for the preparation process of EWZ is shown in Scheme 2b.

### 2.6. Preparation of Waterborne Epoxy Thermoset Composite Coatings Containing 0.25, 0.5, and 0.75% w/w GO with ZD (WGZ), Respectively

A suitable amount of GO dispersed in 1 mL of DI water was introduced into a sample bottle (refer to Table 1). The sample bottle containing GO solution was placed in a shaker and shaken for 3 h, until uniform dispersion was achieved. Subsequently, 0.5 g of EP 147w was introduced into the sample bottle under magnetic stirring for 12 h. Then, EH613w was introduced into the sample bottle under magnetic stirring for an additional 2 h. Next, different weight ratios of ZD were introduced into the sample bottle under magnetic stirring for 6 h. The as-prepared mixing solution was then coated uniformly onto the CRS coupon, followed by placing the CRS coupon into a programmed furnace. After curing, the WETCs containing 0.25, 0.5, and 0.75% *w*/*w* GO with ZD (WGZ) were obtained. The flowchart for the preparation process of WGZ is shown in Scheme 2c.

### 2.7. Preparation of Waterborne Epoxy Thermoset Composite (WETC) Coatings Containing 5% w/w ACAT and 0.5% w/w GO (EWGZ)

WETC coatings containing specific amounts of ACAT and GO with ZD were prepared based on Table 1. The flowchart of the preparation of WETC coatings is illustrated in Scheme II. As an example, the preparation procedure of the E5WG0.5Z35 coating involved several sequential steps. Initially, the dispersion of 0.5% *w*/*w* GO in water was achieved through ultrasonic treatment for 3 h. This was followed by the dissolution of 5% *w*/*w* ACAT in the GO solution under magnetic stirring for 1 h. Subsequently, EH613W was introduced into the solution and stirred magnetically for 24 h. Lastly, 30% *w*/*w* ZD was blended into the epoxy slurry obtained from the previous steps under magnetic stirring for 6 h.

### 2.8. Preparation of WETC Coatings for Electrochemical Corrosion Measurements

First, the coatings were applied separately onto the CRS coupons to create the working electrodes (WEs). To create a 3-electrode system, each of these WEs was used together with the saturated calomel electrode (SCE) and carbon rod. The latter set served as the reference electrode and counter electrode, respectively. Finally, all electrodes were immersed into a 3.5% *w*/*w* NaCl aqueous solution, which functioned as the electrolyte for electrochemical measurements. The results were analyzed using Tafel plots and Electrochemical Impedance Spectroscopy (EIS) to obtain data on the Ecorr, Icorr, and Rp of the materials. The measured data are shown in Table 1.

### 2.9. Preparation of WETC Coatings for Salt Spray Testing

Testing was conducted according to the specifications of the American Society for Testing and Materials (ASTM B-117) [42] and the national standards of Taiwan, including CNS 8886 and Z8026. The test specimens were made of carbon steel with dimensions of 70 mm × 150 mm and a thickness of 145 ± 10 μm. After applying the aforementioned coatings onto the specimens, two parallel cuts, each 40 mm in length, were made along the diagonals of the specimen surface.

### 2.10. Preparation of WETC Coatings for Adhesion Testing and Wear Resistance Testing

The adhesion test followed the ASTM-D3359 standard [43], also known as the “Standard Test Method for Measuring Adhesion by Tape Test”. This method involved measuring the adhesive strength through a tape test. The test entailed creating a grid pattern with six to eleven cuts in each direction across the entire film, applying a specified pressure-sensitive adhesive tape onto the grid, and subsequently removing it. Adhesion was assessed by comparing the results with descriptions and images provided in the standard.

## 3. Results and Discussion

### 3.1. Characterization of Commercial Waterborne Epoxy Thermoset Coating (WETC)

**Figure 1:**
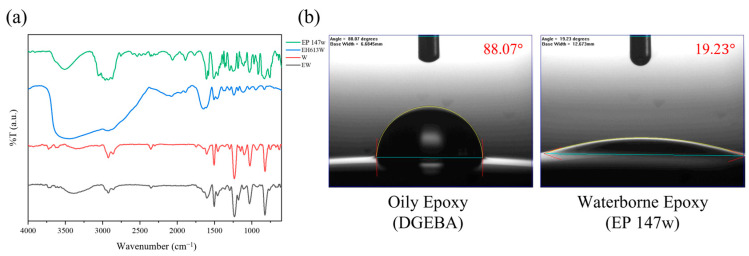
Preliminary identification of commercially available resins: (**a**) FTIR, (**b**) contact angle measurements.

Based on the FTIR spectral analyses, the WETC of EP 147w exhibited a characteristic epoxy signal at ~915 cm^−1^. On the other hand, the curing agent of EH613W revealed an obvious characteristic amine signal at 3000–3500 cm^−1^. After performing the thermal ring-opening polymerization, the characteristic peaks of both epoxy and amine of WET disappeared, indicating that the epoxide ring of EH613W had reacted with the primary group of the hardener, as shown in Figure 1a. On the other hand, 5% *w*/*w* ACAT functioned as a curing agent that partially replaced EH613W. This was evident in the disappearance of the corresponding characteristic primary amine of ACAT and epoxide ring signal of the as-prepared coatings.

For the comparative study of surface wettability, the contact angle of water droplets (CAWD) on the solvent-based epoxy resin (i.e., DGEBA) was found to be 88.07°. However, the CAWD on the waterborne epoxy resin (i.e., EP 147w) was found to be 19.23°, as shown in Figure 1b. It can be concluded that the waterborne epoxy resin of EP 147w exhibited higher hydrophilicity and a lower CAWD.

### 3.2. Characterization of Commercial ZD

**Figure 2:**
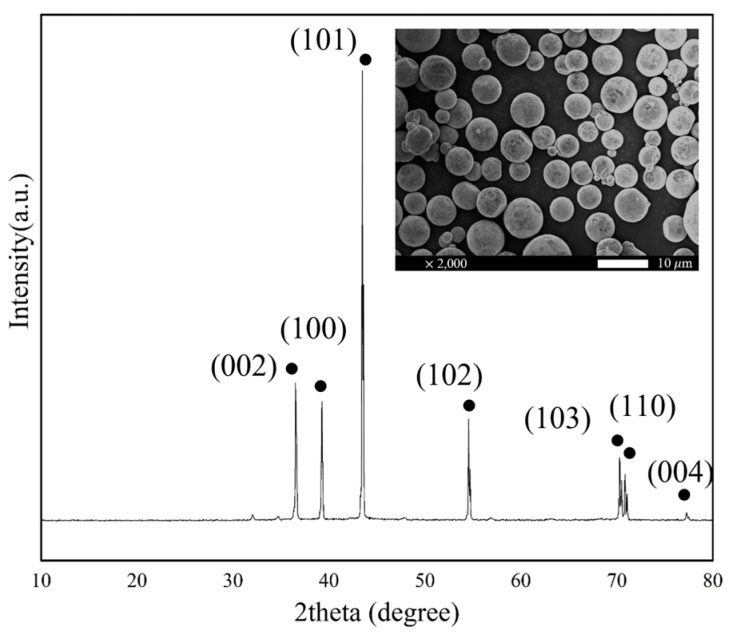
Crystal structure of commercial ZD investigated by XRD (upper right corner is SEM image of commercial ZD).

The crystal structure of the ZD was confirmed through its powder XRD pattern, illustrated in Figure 2, where seven characteristic peaks appeared at 2θ = 36.3, 38.9, 42.3, 54.3, 70.1, 70.6, and 77.0, corresponding to the (002), (100), (101), (102), (103), (110), and (004) planes, respectively [49]. This XRD pattern attests to the purity of the ZD used in the study, indicating no oxidation into ZnO. Additionally, the surface morphology of ZD was examined under a scanning electron microscope at 2000x magnification, revealing particle diameters ranging from 3 to 8 μm, as depicted in Figure 2. The white scale bar in the SEM image denotes 10 μm.

### 3.3. Characterization of ACAT

**Figure 3:**
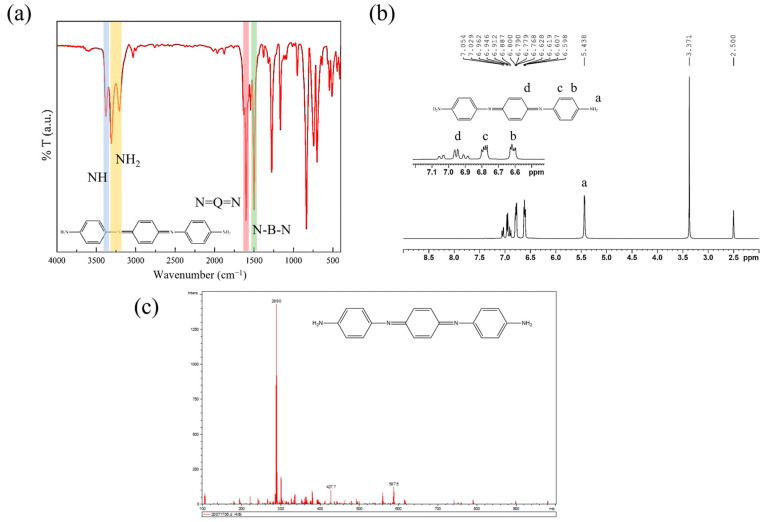
Characterizations of as-synthesized ACAT. (**a**) FTIR spectra, (**b**) ^1^H-NMR spectra, and (**c**) mass spectra.

The procedure for the synthesis of ACAT was recently established by Wei et al., and accordingly, the ACAT could be easily synthesized by oxidative coupling of 1,4-phenylenediamine and two equivalents to aniline with ammonium persulfate as the oxidant [42]. The detailed characterizations of the ACAT are listed as follows: FTIR (KBr, cm^−1^): 3310 and 3200 (s, υ_NH_), 1600 (s, υ_C=C_ of quinoid rings), 1500 (vs, υ_C=C_ of benzenoid rings), 1280 (s, υ_C-N_), 830 (m, para-substitution of benzene ring) [50]. ^1^H NMR (300 MHz, d_6_-DMSO): δ = 6.6–7.0 (multiplet for 12 H from aromatic ring), δ = 5.4 (2H, due to -NH_2_). MS: [M-H]^+^ calculated, 289.0; found, 289.0. The MS, FTIR, and ^1^H NMR spectra of ACAT are shown in Figure 3.

**Figure 4:**
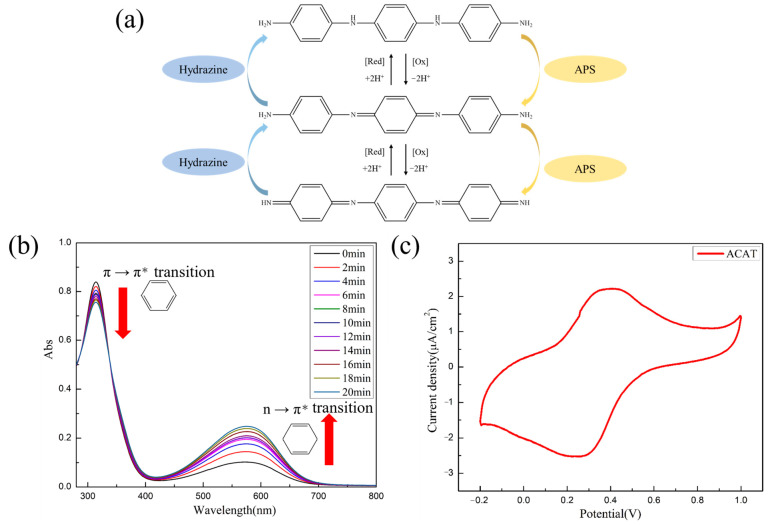
(**a**) Reversible redox behavior of ACAT. (**b**) Reversible doping/de-doping capability of ACAT, monitored by UV-vis absorption spectra. (**c**) Reversible redox capability of ACAT, investigated by electrochemical cyclic voltammetry.

As depicted in Figure 4a, the reversible redox mechanism of aniline oligomer (ACAT) in three distinctive oxidation states can be finely adjusted by adding hydrazine to reduce ACAT or by adding ammonium persulfate (APS) to oxidize ACAT. These alterations can be monitored using a UV-vis spectrophotometer. Figure 4b illustrates that initially, only one characteristic absorption band was present at 330 nm (a band), associated with the π-π* transition of the conjugated ring system of ACAT [51,52]. Upon the slow oxidation of the aniline oligomer (ACAT) following the introduction of a trace amount of ammonium persulfate (APS), the absorption band underwent a blue shift, accompanied by a decrease in intensity. Additionally, a new absorption band (b band) emerged at 590 nm, corresponding to benzenoid and quinoid excitation transitions. As time progressed, the intensity of the b band increased, while its λmax remained constant. This gradual increase in the degree of ACAT oxidation was evident in the strengthening of the quinone ring band. When the intensity of the b band peaked, a blue shift was observed. These findings provide further confirmation of the inherent redox properties of ACAT.

Furthermore, Figure 4c displays the representative electrochemical cyclic voltammetry (CV) of aniline oligomer (ACAT). The CV curve of ACAT exhibits two distinct oxidation peaks and reduction peaks. On the oxidation curve, the first peak is observed at 0.3 V, indicating the conversion or oxidation of the fully reduced state of ACAT to a half-oxidized state. Subsequently, a second oxidation peak appears at approximately 0.5 V, indicating the conversion of ACAT from a half-oxidized state to a fully oxidized state. Conversely, on the reduction curve, two reduction peaks for ACAT are observed at 0.2 V and 0.4 V, signifying the reduction of fully oxidized ACAT to the half-oxidized state, followed by further reduction to the fully reduced state.

### 3.4. Characterization of GO

**Figure 5:**
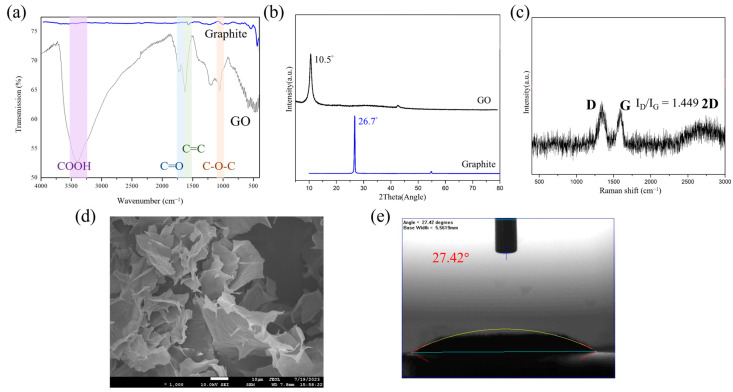
Characterizations of GO: (**a**) FTIR spectra, (**b**) XRD patterns, (**c**) Raman spectra, (**d**) SEM image, (**e**) contact angle of water droplets.

As shown in Figure 5a, compared to graphite, GO exhibits characteristic FTIR peaks at 3420 cm^−1^, 1725 cm^−1^, 1565 cm^−1^, and 1220 cm^−1^, representing the vibrational absorption peaks of O-H, C=O, C=C, and C-O-C functional groups on graphene oxide, respectively. The peak at 1045 cm^−1^ may correspond to the vibrational absorption peak of C-OH or C-O-C. Thus, it can be inferred that the graphite was successfully oxidized to GO after treatment with Hummer’s method.

Figure 5b presents the XRD analysis of GO. It is well established that graphite exhibits a prominent diffraction peak at 2θ = 26.70, corresponding to its characteristic lattice (002) plane, with its surface graphite sheets arranged in an orderly fashion [53]. However, following the oxidation of graphite to GO using Hummer’s method, the distinctive (002) peak vanishes, giving rise to a novel peak at 2θ = 10.50. This shift indicates an increase in the interlayer spacing between graphene platelets, suggesting the formation of oxygen-containing functional groups within the graphene platelet structure.

To delve into the structural properties of the as-prepared GO, Raman spectroscopic analysis was conducted. As depicted in Figure 5c, the D and G bands of GO originated from sp^2^ vibrations within the aromatic ring structure. Generally, the D band corresponds to the vibration of carbon atoms perpendicular to the graphene plane, indicating the presence of defects or edges in the lattice. Conversely, the G band represents the stretching vibration of carbon atoms within the graphene plane [54]. The ratio of the intensities of the D and G bands (I_D_/I_G_) serves as an indicator of practical defects in the GO material. A higher I_D_/I_G_ ratio signifies a more irregular arrangement of the material and a higher degree of defects. In the case of the as-prepared GO, the I_D_/I_G_ value was found to be 1.449.

The microscopic surface pattern of graphene and its surface structure were revealed in the SEM images, as shown in Figure 5d. When graphite was oxidized into GO using Hummer’s method, its surface exhibited a relatively flat lamellar structure. Through the water contact angle, it can be observed that the water contact angle of GO was only 27.42°, indicating that its surface is very hydrophilic. This could be attributed to the surface of GO, which contains many hydrophilic functional groups, such as -COO, C-O-C, and C-OH [55,56].

### 3.5. Characterization of Coatings

**Figure 6:**
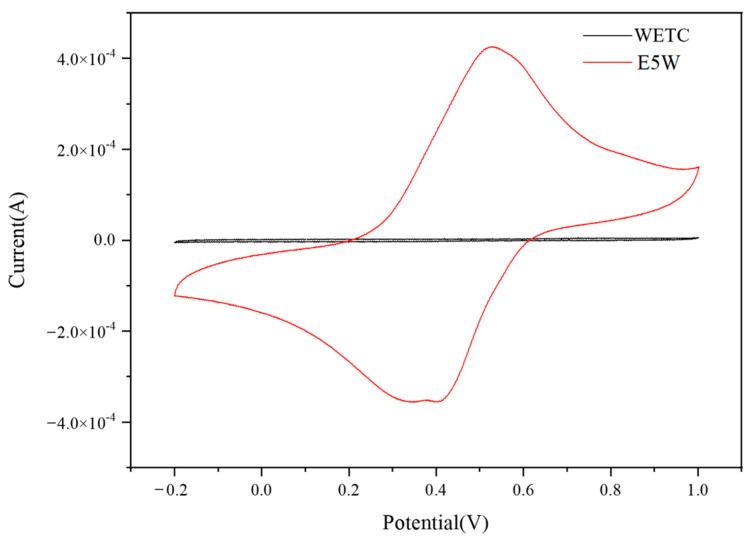
Redox capability of WETC and WETC containing 5% *w*/*w* of ACAT (E5W) measured by electrochemical CV studies.

Electrochemical cyclic voltametric studies were employed to investigate the redox properties of both the WETC and EW. In Figure 6, the CV curve for the WETC reveals a linear profile along the *x*-axis, suggesting the absence of any evident redox behavior in the case of the pure WETC. However, a closer examination of E5W shows oxidation current (I_ox_) and reduction current (I_red_) values of 0.425 mA/cm^3^ and −0.355 mA/cm^3^, respectively, indicating the occurrence of redox behavior in EW.

### 3.6. Potentiodynamic Measurements of Composite Coatings

**Figure 7:**
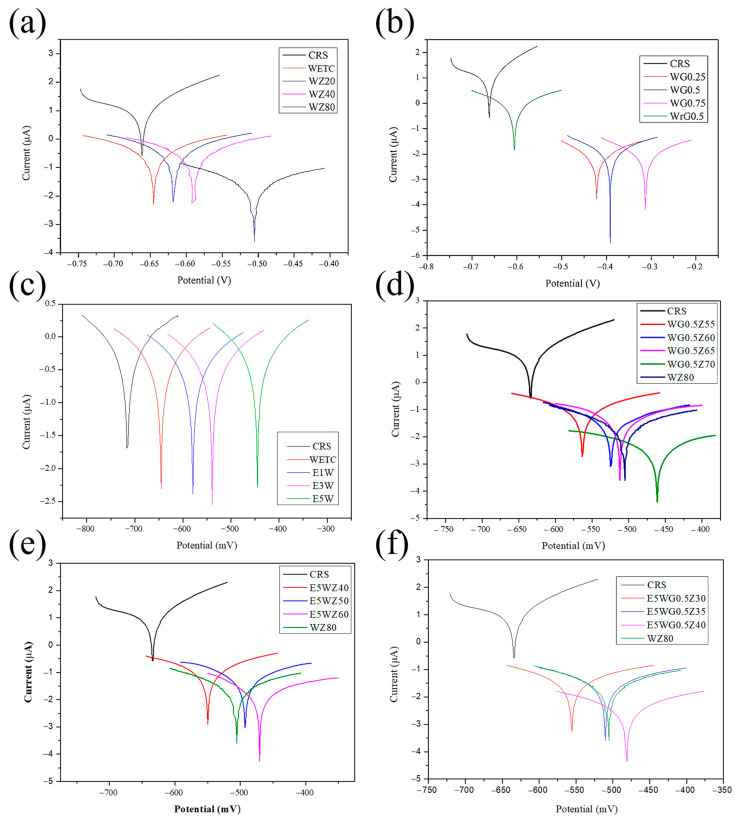
Full immersion of all samples in 3.5% *w*/*w* NaCl aqueous solution for (**a**) Tafel plots of CRS electrode and CRS electrode coated with WETC, WZ20, WZ40, and WZ60. (**b**) Tafel plots of CRS electrode and CRS electrode coated with WG0.25, WG0.5, WG0.75, and WrG0.5. (**c**) Tafel plots of CRS electrode and CRS electrode coated with WETC, E1W, E3W, and E5W. (**d**) Tafel plots of CRS electrode and CRS electrode coated with WG0.5Z55, WG0.5Z60, WG0.5Z65, WG0.5Z70, and WZ80. (**e**) Tafel plots of CRS electrode and CRS electrode coated with E5WZ40, E5WZ50, E5WZ60, and WZ80. (**f**) Tafel plots of CRS electrode and CRS electrode coated with E5WG0.5Z30, E5WG0.5Z35, E5WG0.5Z40, and WZ80.

Figure 7a displays the Tafel plot with ZD content. It can be observed from the figure that as the ZD content increases, the corrosion resistance of the coating improves.

To investigate how the characteristic gas barrier of graphene oxide (GO) influences the anticorrosion performance of the WETC coatings on the CRS electrode, WETC formulations containing varying concentrations of GO (0.25%, 0.5%, and 0.75% *w*/*w*) were prepared. The Tafel plot depicted in Figure 7b illustrates the outcomes, while Table 1 presents the corresponding data. The findings indicate that the well-dispersed GO platelets within the WETC coatings effectively elongate the diffusion pathways for oxygen and reduce the coatings’ permeability to water [57,58]. When the content of graphene oxide (GO) increases to 0.75% *w*/*w*, aggregation begins to occur, leading to a decrease in the corrosion resistance properties of the coating. Therefore, the optimal addition amount of GO is 0.5% *w*/*w*. Additionally, if 0.5% *w*/*w* of reduced graphene oxide (rGO) is added, the corrosion resistance properties deteriorate significantly. This could be attributed to the fewer hydrophilic functional groups that are present in rGO, which hinders its effective dispersion in the waterborne resin.

To analyze the impact of the electrocatalytic properties of the aniline oligomer on the anticorrosion efficacy of epoxy (EW) coatings, a series of electrochemical potentiodynamic measurements were conducted under saline conditions, as depicted in Figure 7c, with the corresponding data summarized in Table 1. The results reveal that an increased loading of aniline oligomer in EW coatings corresponds to heightened electrocatalytic activity, as evidenced by the electrochemical cyclic voltammetry (CV) curve. Consequently, this augmentation promotes the overall anticorrosion performance of the coating. This mechanism underscores how the electroactive E5W coating, with its enhanced anticorrosion effect compared to the WETC coating, may be attributed to the electrocatalytic properties of aniline oligomer inducing the formation of a densely passivated metal oxide layer, thereby shielding the underlying metallic substrate [59,60].

The data presented in Figure 7d suggest that both WG0.5Z65 and WZ80 coatings demonstrate nearly identical E_corr_ and I_corr_ values, implying comparable anticorrosion performance. This implies that an epoxy composite coating containing 0.5% *w*/*w* graphene oxide (GO) can effectively substitute 15% *w*/*w* zinc dust (ZD). Additionally, Figure 6e illustrates that E5WZ50 and WZ80 coatings offer similar corrosion protection, indicating that a coating comprising 5% *w*/*w* aniline oligomer (ACAT) can replace 30% *w*/*w* ZD.

To further minimize the usage of ZD in coatings, a series of composites were developed by incorporating the electrocatalytic properties of ACAT and the characteristic gas barrier of GO into the coatings, as illustrated in Figure 7f. Notably, the E5WG0.5Z35 and WZ80 coatings demonstrated comparable corrosion protection, indicating that coatings formulated with 5% *w*/*w* ACAT and 0.5% *w*/*w* GO can effectively substitute 45% *w*/*w* ZD.

Open circuit potential (OCP) serves as a valuable tool for understanding the protection mechanism in metal corrosion protection electrochemistry. The “self-corrosion potential,” as measured by the Tafel curve, reflects the resting potential of the electrode, indicating the point at which external current ceases. Thus, at the system’s equilibrium, the OCP corresponds to the corrosion potential (E_corr_ in V vs. SCE) [61]. Consequently, corrosion potential data derived from Tafel tests provide an insight into the protection mechanism.

An examination of the corrosion potential data in Table 1 reveals that the corrosion potentials of all coatings surpass the cathodic protection potential limit of −760 mV [62]. This observation suggests that zinc dust (ZD) is effectively shielded by uniformly dispersed graphene oxide (GO) nanosheets, which act as impermeable physical barriers. Therefore, it can be inferred that during the immersion test period, the prepared coatings serve as physical barriers, providing a shielding effect for the metal substrate.

Table 2 presents a comparative analysis between the current study and prior research endeavors conducted by both our team and other researchers. Over the past few years, our team has dedicated efforts to refining the composition of coatings. In this study, a novel approach is introduced by utilizing small quantities of graphene oxide (GO) and aniline oligomer (ACAT) to substitute a substantial amount of zinc dust. This unprecedented achievement represents a significant departure from previous methodologies. It is worth noting that many previous studies by other researchers have employed a 1:1 or 1:2 ratio to replace zinc dust. For instance, Professor B. Ramezanzadeh respectively utilized 10 wt% of micaceous iron oxide and Al to substitute for 10 wt% of ZD [17], or 2 wt% of modified nano-aluminum powder to replace 2 wt% of ZD [63]. Professor Hao Wu respectively employed 2.5, 5, and 10 wt% of stainless steel flakes to replace double the amount of ZD [64]. These approaches are neither cost-effective nor associated with superior performance.

### 3.7. Salt Spray Test of CS Coated with Epoxy, WGZ, or EWGZ

**Figure 8:**
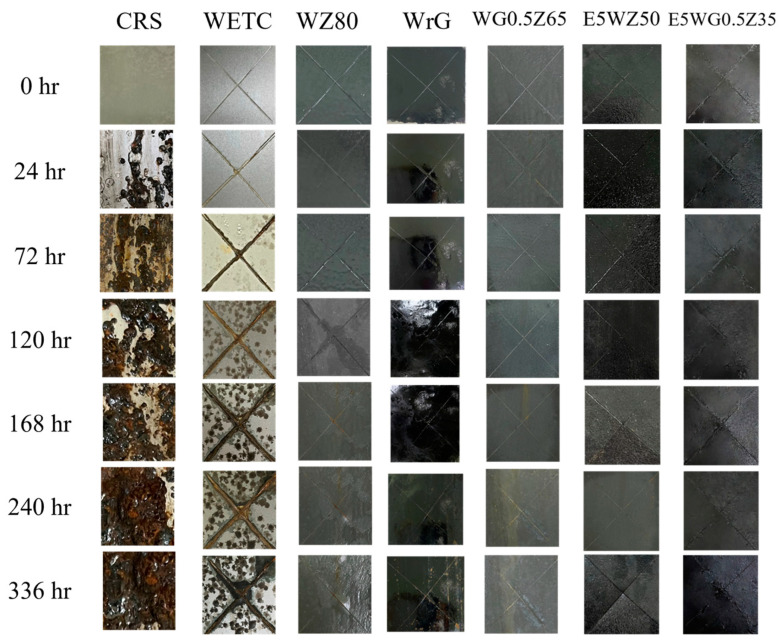
Salt spray testing of raw CRS and CRS coated with distinctive epoxy-derived coatings (i.e., WETC, WZ80, WrG, WG0.5Z65, E5WZ50, and E5WG0.5Z35).

The photographs in Figure 8 illustrate the surface conditions observed during accelerated salt spray tests conducted on CS samples, both with and without coatings, at various testing intervals (24, 72, 120, 168, 240, and 336 h). In the case of untreated CS exposed to salt spray testing, noticeable natural oxidation occurred. After 72 h, corrosion was evident on the WETC coating, while the remaining coatings remained corrosion-free. Notably, WZ80 exhibited effective corrosion protection, even after 336 h of salt spray testing, based on visual inspection. During the evaluation of aniline oligomer (ACAT)’s corrosion protection performance, the E5WZ50 coatings also demonstrated good corrosion resistance after 120 h of testing. Additionally, in the study of GO’s corrosion protection ability, the WG0.5Z65 coating exhibited effective corrosion protection even after 168 h. When considering coatings that combine the electrocatalytic attributes of ACAT with the gas barrier properties of GO, the E5WG0.5Z35 coatings provided reliable corrosion protection throughout the entire 336 h salt spray test.

In summary, after 336 h of salt spray testing, WZ80, E5WZ50, WG0.5Z65, and E5WG0.5Z35 demonstrated commendable corrosion protection. This outcome was consistent with the previous conclusion drawn from the electrochemical corrosion measurements.

### 3.8. Adhesion of the Coatings

**Figure 9:**
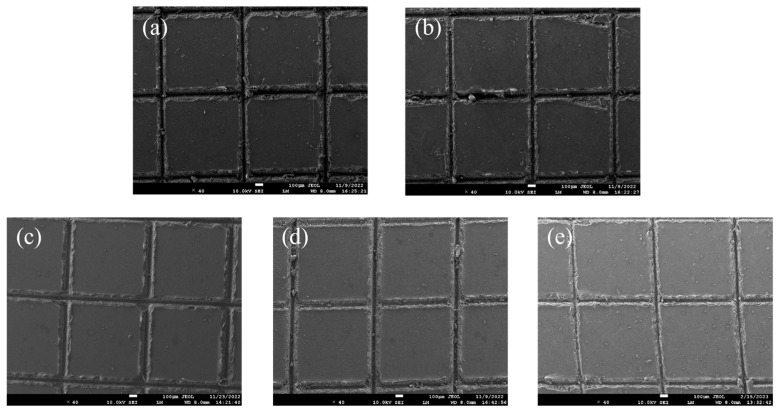
SEM images obtained from the adhesion testing of distinctive epoxy-derived coatings (**a**) WETC, (**b**) WZ80, (**c**) WG0.5Z65, (**d**) E5WZ50, and (**e**) E5WG0.5Z35.

According to ASTM 3359 standards, the adhesion of the coatings prepared in this study underwent testing. Figure 9 and Table 1 showcase SEM images following cross-cutting and the application of Scotch tape to the coatings. In Figure 9a, the SEM image of the WETC coating reveals strong adhesion to the substrate, with no signs of peeling. The edges of the cut in the WETC coating are entirely smooth, devoid of any flaking at the lattice edges, resulting in an adhesion rating of 5B. Conversely, in Figure 9b, the SEM image of WZ80 indicates that a portion of the incision edge has peeled off, with the peeled area measuring less than 5%. Consequently, the adhesion rating for the WZ80 coating was classified as 4B. The WG0.5Z65 coating also received an adhesion rating of 5B, as observed in Figure 9c. Moving on to Figure 9d,e, which showcase the SEM images of the cross-cuts for the E5WZ50 and E5WG0.5Z35 coatings, all these samples exhibited coatings with robust integrity. The cutting edges remained entirely smooth, devoid of any flaking, resulting in an adhesion rating of 5B for each of these coatings.

Due to the limited conductivity of the polymer used as an adhesive, a significant amount of ZD (above 80 wt.%) is required to ensure electrical continuity for the desired electrochemical reactions, both among the zinc particles themselves and with the steel substrate. However, the high loading of ZD leads to reduced coating flexibility and weak adhesion to the steel substrate. Adhesion tests revealed that the coating with 80 wt.% ZD (WZ80) achieved an ASTM grade of 4B. By reducing the zinc powder content to 65, 50, and 35 wt.%, respectively, through the addition of GO and ACAT, the adhesion of the coatings was improved to a grade of 5B.

### 3.9. Abrasion of Coatings

**Figure 10:**
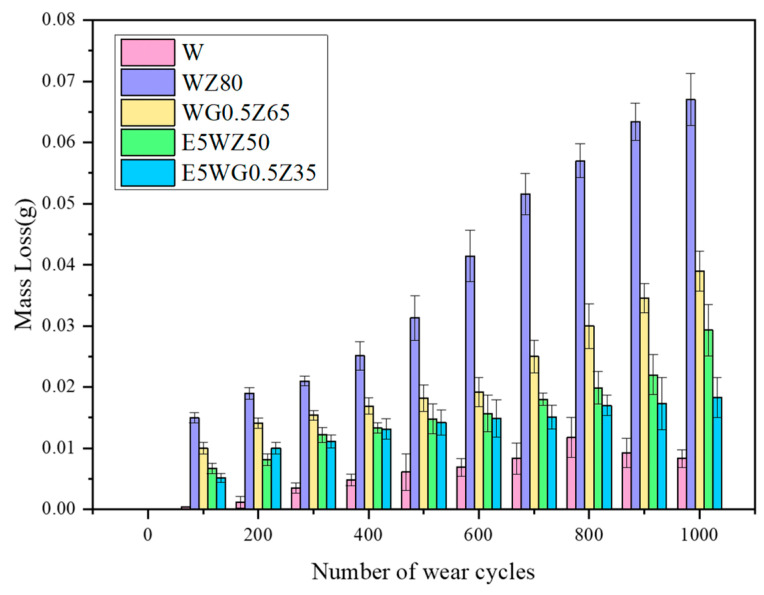
Mass loss obtained from the wear resistance testing of CRS coated with distinctive epoxy-derived coatings (i.e., WETC, WZ80, WG0.5Z65, E5WZ50, and E5W0.5RZ35).

A series of coatings underwent preparation and subsequent wear resistance testing in accordance with the ASTM D4060 standards. The relationship between mass loss and the number of wear cycles is illustrated in Figure 10, with corresponding data provided in Table 1. The W coating exhibited a mass loss of 0.0130 g after 1000 wear cycles. However, upon introducing 80% *w*/*w* ZD, the resulting WZ80 coating displayed an increased mass loss of 0.0670 g after 1000 wear cycles. This suggests that the incorporation of ZD led to a decrease in the wear resistance of the coating. In contrast, the WG0.5Z65 coating, which contains ZD and GO, exhibited a mass loss of 0.0390 g after 1000 wear cycles. Incorporating 0.5% *w*/*w* GO may enhance the wear resistance of the coating while preserving its corrosion protection capabilities. Moving on to the E5WZ50 coating, containing ZD and ACAT, it displayed a mass loss of 0.0293 g after 1000 wear cycles. This signifies that the incorporation of 5% *w*/*w* ACAT effectively improved the wear resistance of the E5WZ50 coating while maintaining corrosion protection levels that were similar to those of the WZ80 coating. This result suggests that a higher ACAT content and lower ZD content contribute to enhanced wear resistance in the composite coating. Lastly, considering the wear resistance of the E5WG0.5Z35 coating, which includes ZD, ACAT, and GO, the mass loss after 1000 wear cycles was merely 0.0183 g. This indicates that introducing 5% *w*/*w* ACAT and 0.5% *w*/*w* GO into the WZ35 coating significantly enhanced its wear resistance without compromising its corrosion protection, compared to the WZ80 coating. These findings underscore the positive impact of combining 0.5% *w*/*w* GO, 5% *w*/*w* ACAT, and a reduced ZD content on promoting the wear resistance of the composite coating.

## 4. Conclusions

In this study, a series of waterborne zinc-rich epoxy coatings containing aniline oligomer (ACAT) and/or graphene oxide (GO) were developed and applied as heavy-duty anticorrosion coatings. These coatings effectively reduced zinc dust (ZD) loading while maintaining their anticorrosion performance and enhancing adhesion and wear resistance. Electrochemical corrosion measurements revealed that the incorporation of 80% *w*/*w* ZD significantly enhanced the corrosion protection of the coating, as evidenced by a shift in the corrosion potential (−506.4 mV) and a decrease in the corrosion current density (0.0124 µA/cm^2^) compared to neat epoxy coatings (−645.6 mV and 0.189 µA/cm^2^, respectively).

Furthermore, the electrochemical measurements of the coatings containing either ACAT or GO demonstrated a rightward shift of the Tafel curve, suggesting an enhanced protection capability with the incorporation of these additives. ACAT’s electrocatalytic characteristic facilitated the formation of a densely passivated metal oxide layer, effectively shielding the underlying metallic substrate. Additionally, the well-dispersed graphene oxide (GO) platelets within the epoxy matrix enhanced the gas barrier property of the corresponding coating.

This study also explored the potential for reducing ZD loading by replacing it with ACAT and GO. The findings indicated that the simultaneous addition of 5% *w*/*w* ACAT and 0.5% *w*/*w* GO could potentially replace 45% *w*/*w* ZD while maintaining similar corrosion protection performance.

In terms of future research, there is ample opportunity to optimize composition and processing parameters, aiming to enhance coating performance. Delving into the synergistic interactions between ACAT, GO, and ZD could yield insights to bolster corrosion protection. Advanced techniques like in situ spectroscopy could provide deeper insights into structure–property relationships.

From a market perspective, the incorporation of GO and ACAT as a replacement for zinc dust presents a compelling advantage. This innovative approach not only reduces the environmental impact but also offers enhanced corrosion protection properties. By emphasizing these benefits and conducting extensive field testing, these coatings can secure their position across various sectors like marine, automotive, aerospace, and infrastructure, ensuring commercial success and widespread societal impact.

## Figures and Tables

**Table 1:** Electrochemical corrosion measurements, adhesion testing, and wear resistance testing of the as-prepared distinctive epoxy-derived coatings.

Sample Code	Composition				Electrochemistry			Thickness (µm)	Adhesion Test ^1^	Mass Loss of Wear Test (g) ^2^
	Epoxy (wt%)	ZD (wt%)	ACAT (wt%)	GO (wt%)	Ecorr (mV)	Icorr (µA/cm^2^)	PEF (%)			
CRS	-	-	-	-	−660.1	27.0	-	-	-	-
WETC	100	-	-	-	−645.6	0.189	99.30	150 ± 1.7	5B	0.0130
WZ80	20	80	-	-	−506.4	0.0124	99.95	150 ± 2.2	4B	0.0670
WG0.5Z65	34.5	65	-	0.5	−511.9	0.0116	99.95	151 ± 2.1	5B	0.0390
E5WZ50	45	50	5	-	−496.8	0.00808	99.97	147 ± 1.1	5B	0.0293
E5WG0.5Z35	59.5	35	5	0.5	−510.3	0.00120	99.96	149 ± 1.7	5B	0.0183

^1^ Determined by adhesion test based on ASTM D3359. ^2^ Determined by wear test based on ASTM D4060.

**Table 2:** This study compares data with relevant literature on zinc powder substitution.

	Researcher	Compound Used to Replace ZD	Compound Ratio (wt%)	ZD Replaced (wt%)	Ref.
1	Carlos Alemán (2010)	polyaniline emeraldine salt	0.3	19	[65]
2	B. Ramezanzadeh (2013)	micaceous iron oxide (MIO)	10	10	[17]
		Al	10	10	
		MIO + Al	10 (5 + 5)	10	
3	B. Ramezanzadeh (2015)	modified nano-aluminum powder	2	2	[63]
4	Hao Wu (2022)	stainless steel flakes (SSFs)	2.5	5	[64]
			5	10	
			10	19	
5	Jui-Ming Yeh (2023)	300 °C rGO	0.5	20	[40]
		1400 °C rGO	0.5	30	
6	Jui-Ming Yeh (2023)	rGO	0.5	20	[41]
		aniline pentamer-based diamine (DAAP)	1	30	
			5	50	
		rGO + DAAP	1.5 (0.5 + 1)	50	
			5 (0.5 + 5)	70	
7	This study	GO	0.5	15	-
		ACAT	5	30	
		GO + ACAT	5.5 (0.5 + 5)	45	

## Data Availability

Data are contained within the article.
